# Modulating Effects of Cancer-Derived Exosomal miRNAs and Exosomal Processing by Natural Products

**DOI:** 10.3390/cancers15010318

**Published:** 2023-01-03

**Authors:** Ya-Ting Chuang, Jen-Yang Tang, Jun-Ping Shiau, Ching-Yu Yen, Fang-Rong Chang, Kun-Han Yang, Ming-Feng Hou, Ammad Ahmad Farooqi, Hsueh-Wei Chang

**Affiliations:** 1 Graduate Institute of Medicine, College of Medicine, Kaohsiung Medical University, Kaohsiung 80708, Taiwan; 2 School of Post-Baccalaureate Medicine, Kaohsiung Medical University, Kaohsiung 80708, Taiwan; 3 Department of Radiation Oncology, Kaohsiung Medical University Hospital, Kaoshiung Medical University, Kaohsiung 80708, Taiwan; 4 Division of Breast Oncology and Surgery, Department of Surgery, Kaohsiung Medical University Hospital, Kaohsiung Medical University, Kaohsiung 80708, Taiwan; 5 School of Dentistry, Taipei Medical University, Taipei 11031, Taiwan; 6 Department of Oral and Maxillofacial Surgery, Chi-Mei Medical Center, Tainan 71004, Taiwan; 7 Graduate Institute of Natural Products, Kaohsiung Medical University, Kaohsiung 80708, Taiwan; 8 Department of Biomedical Science and Environmental Biology, College of Life Science, Kaohsiung Medical University, Kaohsiung 80708, Taiwan; 9 Institute of Biomedical and Genetic Engineering (IBGE), Islamabad 54000, Pakistan; 10 Institute of Medical Science and Technology, National Sun Yat-sen University, Kaohsiung 80424, Taiwan; 11 Center for Cancer Research, Kaohsiung Medical University, Kaohsiung 80708, Taiwan

**Keywords:** exosome, miRNA, cell function, natural product

## Abstract

**Simple Summary:**

Cancer cells generate exosomes (extracellular vesicles) to regulate many cell functions for tumor progression. Many exosome-modulating clinical drugs have been developed for effective cancer therapy, but the functions and exosome processing (secretion and assembly) modulation by natural products are not well understood. In this review, we fill the gaps between natural products-modulated miRNAs and exosome-processing by the target gene prediction of the bioinformatics database. The cancer-derived exosomal miRNAs and their exosome processing and modulated cell functions by natural products are well organized. Consequently, this review provides a comprehensive and potential modulating mechanism and targets for exosome processing and cancer cell functions for natural products.

**Abstract:**

Cancer-derived exosomes exhibit sophisticated functions, such as proliferation, apoptosis, migration, resistance, and tumor microenvironment changes. Several clinical drugs modulate these exosome functions, but the impacts of natural products are not well understood. Exosome functions are regulated by exosome processing, such as secretion and assembly. The modulation of these exosome-processing genes can exert the anticancer and precancer effects of cancer-derived exosomes. This review focuses on the cancer-derived exosomal miRNAs that regulate exosome processing, acting on the natural-product-modulating cell functions of cancer cells. However, the role of exosomal processing has been overlooked in several studies of exosomal miRNAs and natural products. In this study, utilizing the bioinformatics database (miRDB), the exosome-processing genes of natural-product-modulated exosomal miRNAs were predicted. Consequently, several natural drugs that modulate exosome processing and exosomal miRNAs and regulate cancer cell functions are described here. This review sheds light on and improves our understanding of the modulating effects of exosomal miRNAs and their potential exosomal processing targets on anticancer treatments based on the use of natural products.

## 1. Introduction

Exosomes are extracellular vesicles of 30–100 nm in size that are secreted by both cancer and normal cells [1,2]. Cancer cells secrete more abundant and complex compositions in exosomes than normal cells. Cancer-derived exosomes exhibit diverse functions in regulating proliferation, migration, invasion, metastasis, drug resistance, inflammation, and immune responses [1,3,4].

**Figure 1:**
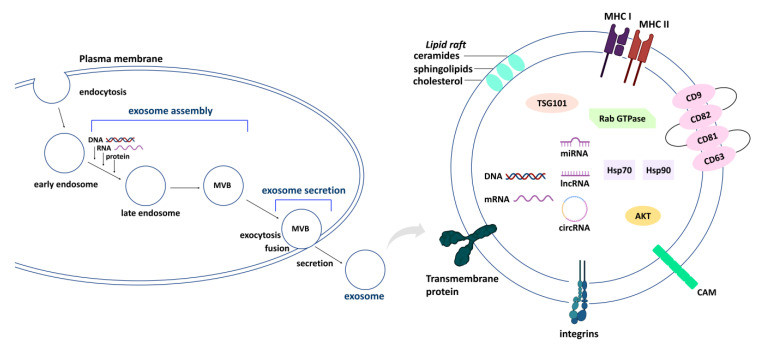
General structure and biogenesis of exosomes. The basic steps of exosome biogenesis, consisting of two main stages, are provided: exosome assembly and secretion. Exosomes generally contain nucleic acids, membrane and soluble proteins, and lipids. Different cells or treatments may have different compositions of exosomes. MVB, multivesicular body.

The general structure and biogenesis of exosomes are shown in Figure 1 [5,6,7,8,9,10,11,12,13]. Many proteins, lipids, DNAs, mRNAs, and non-coding RNAs (circular RNAs, long-noncoding RNAs, and microRNAs (miRNAs)) exist in exosomes [1,2,3]. In general, the membrane proteins of exosomes include major histocompatibility complex (MHC)-I, MHC-II, flotillin, tetraspanins (CD9, CD82, CD81, and CD63), cell adhesion molecules (CAMs), integrins, and transmembrane proteins. The soluble proteins of exosomes include TSG101, heat shock protein 70 (Hsp70), Hsp90, and protein kinase B (AKT). The lipid rafts of exosomes include ceramides, sphingolipids, and cholesterol [5,6,7,8,9,10,11,12,13]. The process of exosome biogenesis starts with the initiation of endocytosis, early endosome, late endosome, and multivesicular body (MVB) formation, plasma membrane fusion, and release by exocytosis [5,6,7,8,9,10,11,12,13]. Exosome biogenesis consists of two stages, namely exosomal assembly and secretion. Many exosomal components (DNA, RNA, and proteins) are uploaded during exosome biogenesis.

miRNAs are a group of small non-coding RNAs of 21–25 nucleotides in size. miRNAs can modulate gene expression by inhibiting mRNA translation or improving the mRNA degradation of target genes [14]. By binding to the 3′-untranslated regions (UTR) of target genes, miRNAs can knock down target gene expressions to assess their diverse functions.

Among the non-coding RNAs, this review focuses only on miRNAs, particularly exosomal miRNAs. miRNA uptake into exosomes is not a random but selective process involving secretion and transportation between exosome donors and receptors [15]. Exosomes play a vital role in regulating the development of oral [16,17], head/neck [18], breast [19], prostate [20], pancreatic [21], colon [22], gynecologic [23], liver [24], and myeloma cancer cells. In preclinical applications, exosomes are applied in diagnosis as several cancer biomarkers [25,26] and in cancer therapy using animal models [27,28,29,30]. Moreover, the exosomal miRNAs also function as modulators of drug resistance and cancer metastasis [25].

As mentioned above, exosomes and miRNAs have a close relationship in regulating cell functions. Recently, anticancer studies using natural products have shown progression in research involving exosomes and miRNAs. However, the potential impacts of exosomes and miRNAs on natural-product-regulating cancer cell functions lack systemic organization. The modulating effects of natural products on exosome biogenesis and exosomal miRNAs are discussed later, particularly in regard to their capacity for regulating exosomal processing (secretion and assembly). Moreover, some natural products and exosomal miRNAs show anticancer effects but lack investigation regarding their impacts on exosomal processing. This gap can be filled by utilizing the miRDB database [31], a bioinformatic tool which can predict the target genes of exosome processing by inputting natural-product-modulated exosomal miRNAs.

**Figure 2:**
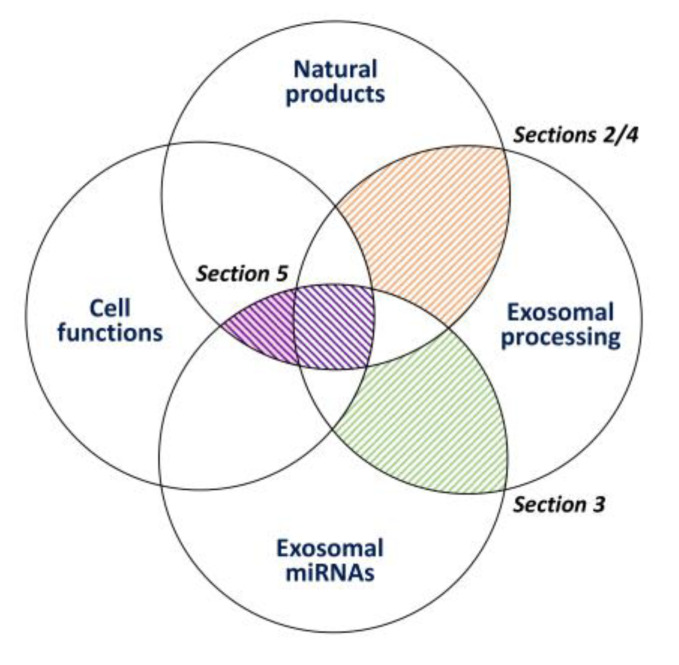
Connections between different sections of this review. Connections between natural products and exosomal processing are examined in Section 2 and Section 4. The connection between exosomal processing and exosomal miRNAs is examined in Section 3. Finally, connections between cancer cell functions, exosomal miRNAs, and natural products are examined in Section 5.

In the following review, we first explore the relationship between exosome processing (secretion and assembly) and natural products (Section 2), because the impact of exosome processing is rarely discussed in detail in the literature. Next, the prediction of the targeting of exosome-processing and AKT-signaling genes of exosome miRNAs is assessed (Section 3), because the contribution of exosome processing is rarely emphasized in the literature. The modulating effects of exosome production by natural products and their exosome delivery potential for cancer treatment (Section 4) are explored. Finally, the regulation of the cancer cell functions of natural-product-modulating miRNAs and exosomes (Section 5) is summarized (Figure 2). Consequently, this review sheds light on the organization of the relationship between exosomal processing and its related genes, exosomal miRNAs, cell functions, and natural products.

## 2. Exosome Processing (Secretion and Assembly) and Natural-Product-Modulated Cell Functions

### 2.1. Exosome Processing (Secretion and Assembly) Genes

Several extracellular biogenesis genes, including exosomal secretion and assembly, were summarized using the Mouse Genome Database in Gene Oncology (GO) functions http://www.informatics.jax.org/vocab/gene_ontology/GO:1990182 (retrieval date: 11 November 2022) [32]. Exosomal secretion begins with the fusion of the partial endosomal membrane of a multivesicular body (MVB) with the plasma membrane, and it ends with the release of membrane-bounded vesicles into the extracellular space (Figure 1). Three main functions of exosomal secretion are classified: multiple vesicular body fusion into the apical plasma membrane, the negative regulation of exosomal secretion, and the positive regulation of exosomal secretion. Furthermore, exosomal assembly is the process in which a set of components are incorporated, aggregated, and bonded to generate an extracellular vesicular exosome. Three main functions of exosomal assembly are classified: extracellular exosome assembly, the positive regulation of extracellular exosome assembly, and the negative regulation of extracellular exosome assembly.

For exosomal secretion, “multiple vesicular body fusion into the apical plasma membrane” includes four genes, including COP9 signalosome subunit 5 (COPS5), RAB11A, a member of the RAS oncogene family (RAB11A), RAB27A, a member of the RAS oncogene family (RAB27A), and STEAP family member 3 (STEAP3). Five genes are listed as modulators of “negative regulation of exosomal secretion”, including ATPase class II, type 9A (ATP9A), parkin RBR E3 ubiquitin protein ligase (PRKN), RAB7A, a member of the RAS oncogene family (RAB7A), RAB7B, a member of the RAS oncogene family (RAB7B), and vacuolar protein sorting 4B (VPS4B).

For exosomal secretion, 14 genes are listed as modulators of the “positive regulation of exosomal secretion”, including ATPase type 13A2 (ATP13A2), charged multivesicular body protein 2A (CHMP2A), HGF-regulated tyrosine kinase substrate (HGS), myosin VB (MYO5B), programmed cell death 6 interacting protein (PDCD6IP), RAB7A, RAB7B, syndecan 1 (SDC1), syndecan 4 (SDC4), syndecan binding protein (SDCBP), sphingomyelin phosphodiesterase 3, neutral (SMPD3), SNF8, the endosomal sorting complexes required for transport (ESCRT)-II complex subunit, homolog (*S. cerevisiae*) (SNF8), signal-transducing adaptor molecule (SH3 domain and ITAM motif) 1 (STAM), tumor susceptibility gene 101 (TSG101), vacuolar protein sorting 4A (VPS4A), and VPS4B. RAB7A, RAB7B, and VPS4B also belong to the genes with a “negative regulation of exosomal secretion” function.

For exosomal assembly, the CD34 antigen (CD34) gene is listed among the cells that affect “extracellular exosome assembly”. Four genes are listed as modulators of “positive regulation of extracellular exosome assembly”, including PDCD6IP, SDC1, SDC4, and SDCBP, which also belong to the group of genes with an exosomal secretion function. Three genes are listed among the cells that affect the “regulation of extracellular exosome assembly”, including PDCD6IP, STAM, and TSG101, which also belong to the group of cells with an exosomal secretion function mentioned above.

### 2.2. Exosomal Secretion and Assembly Effects of Natural Products in Regulating Cell Functions

Some of the exosomal secretion (Section 2.2.1) and assembly (Section 2.2.2) genes have been reported to be regulated by natural products (Table 1).

#### 2.2.1. Exosomal Secretion Effects of Natural Products in Regulating Cell Functions

Natural-product-derived exosomes exhibit a capacity for the sophisticated regulation of cell functions in non-cancer and cancer cells. The potential roles of exosome-processing genes in natural product treatments can be predicted by target gene retrieval using the miRDB database (Table 1).

For non-cancer cells, several natural products exhibit modulating effects on cell functions in regard to exosome processing. Drug-triggered hepatic precancerous lesions upregulate exosomal RAB11A mRNA. Hesperidin downregulates exosomal RAB11A mRNA and upregulates exosomal miR-1298, resulting in hepatoprotective effects on rats (Table 1) [33]. Several natural products, such as tenuifolin, schisandrin A, celastrol, salidroside, and carnosic acid, were demonstrated to exhibit neuroprotection effects through PINK1 modulation [34]. Moreover, PINK1 and PRKN cooperate to regulate the mitophagy of renal proximal tubular cells [35]. Hence, these natural products (tenuifolin, schisandrin A, celastrol, salidroside, and carnosic acid) may modulate PRKN expression. Salvianolic acid B, a *Salvia miltiorrhiza* Bge-derived bioactive compound, suppresses renal interstitial fibrosis by inducing SDC1/E-cadherin in angiotensin II-treated proximal tubular cells [36]. Combined, *Echinacea angustifolia* DC. and *Zingiber officinale* lipophilic extracts show immunomodulatory effects by downregulating SDCBP expression based on human studies [37]. Bavachinin, a *Fructus psoraleae*-derived natural product, provides protection against the palmitic-acid-induced death of hepatocytes by upregulating VPS4B expression (Table 1) [38].

**Table 1:** Connecting natural products to exosomal secretion and assembly in the regulation of cell functions.

Natural Products	Genes	Cell Functions	Cancer	References
**Exosomal secretion**				
Methanolic extract of	↓COPS5	apoptosis	cervical	[39]
*Moringa oleifera*				
Rutin	↓COPS5	apoptosis	cervical	[40]
Hesperidin	↓RAB11A	hepatoprotective	(rat)	[33]
Heteronemin	↑STEAP3	ferroptosis	pancreatic	[41]
Dihydroartemisinin	↓STEAP3	anti-iron uptake	liver	[42]
Robustaflavone A	↓STEAP3	ferroptosis	breast	[43]
Tenuifolin, Schisandrin A,	↑PRKN	mitophagy	(renal tubular cells)	[35]
Celastrol, Salidroside,				
Carnosic acid				
Liensinine	↑RAB7A	anti-autophagy	breast	[44]
Sulfisoxazole	↓VPS4B	antimetastatic	breast	[45]
Bavachinin	↑VPS4B	pro-survival	(hepatocyte)	[38]
Squalamine	↑ATP13A2	α-synuclein aggregation	neuroblastoma	[46]
7-α-Hydroxyfrullanolide	↑PDCD6IP	apoptosis	breast	[47]
Salvianolic acid B	↓SDC1	renal interstitial fibrosis	(proximal tubular cells)	[36]
Rutaecarpine	↓SDC1	antimigration	glioblastoma	[48]
*Echinacea angustifolia*/	↓SDCBP	immunomodulation	(human study)	[37]
*Zingiber officinale*				
extracts				
Dioscin	↑SDCBP	apoptosis, autophagy,	liver ca	[49]
		DNA damage		
Sulforaphane	↑SDCBP	apoptosis	leukemia	[50]
Acetyl-11-keto-b-boswellic	↑SMPD3	antiproliferation	colon	[51]
acid				
Withanolide D	↑/↓SMPD3	apoptosis	leukemia	[52]
Resveratrol	↑TSG101	antiproliferation	intestinal tumor	[53]
**Exosomal assembly**				
*Astragalus membranaceus*	↑CD34	angiogenesis	(myocardial infarction)	[54]
extract				
D Rhamnose bhederin	↓STAM	chemoresistance	breast	[55]

↑, enhance or activate; ↓, inhibit or inactivate. Some are non-cancer studies, shown in parentheses. PDCD6IP, SDC1, SDC4, SDCBP, STAM, and TSG101 exhibit the regulating functions of both exosomal secretion and assembly, as described in Section 2.1.

For cancer cells, several natural products exhibit modulating effects on cell functions in regard to exosome processing. The methanolic extract of *Moringa oleifera* leaves suppresses the proliferation and causes the G1 arrest and apoptosis of cervical cancer cells by downregulating COPS5 (Table 1) [39]. Rutin, a bioflavonoid, induces the apoptosis of cervical cancer cells by downregulating COPS5 [40]. Heteronemin, a marine sesterterpenoid, induces ferroptosis by upregulating the protein expression of divalent metal transporter-1 (DMT1) and STEAP3 in pancreatic cancer cells [41]. Dihydroartemisinin, a metabolite of artemisinin, downregulates the DMT1 and STEAP3 genes controlling iron uptake in liver cancer HepG2 cells (Table 1) [42]. 

*Selaginella trichoclada*-derived robustaflavone A induces ferroptosis by downregulating the expressions of acyl-CoA synthetase long-chain family member 4 (ACSL4), ACSL5, STEAP3, lysophosphatidylcholine acyltransferase (LPCAT3), and autophagy-related 7 (ATG7) genes in breast cancer cells (Table 1) [43]. Liensinine, a *Nelumbo nucifera*-derived isoquinoline alkaloid, induces the expression of the small GTP-binding protein RAB7A and suppresses autophagosome–lysosome fusion for the degradation of breast cancer cells [44]. Squalamine inhibits the α-synuclein aggregation of neuroblastoma cells [56]. The inhibition of ATP13A2 destroys lysosomal membrane integrity and induces the α-synuclein accumulation of neuroblastoma cells (Table 1) [46]. All this warrants a detailed assessment of the role of ATP13A2 in squalamine treatment.

Sulfisoxazole shows antimetastatic effects on breast cancer cells by downregulating VPS4B mRNA expression (Table 1) [45]. 7-α-Hydroxyfrullanolide, an Asteraceae-plant-derived natural product, upregulates PDCD6IP expression in breast cancer cells [47]. Rutaecarpine downregulates the mRNA expression of the SDC1 gene to suppress glioblastoma cell migration [48]. Dioscin, a steroid saponin, causes the apoptosis, autophagy, and DNA damage of liver cancer cells by upregulating SDCBP expression [49]. Sulforaphane, a cruciferous-vegetable-derived compound, triggers apoptosis by upregulating SDCBP expression in leukemia HL-60 cells [50]. Acetyl-11-keto-b-boswellic acid (AKBA), a *Boswellia serrata*-derived natural product, inhibits the proliferation of colon cancer cells by upregulating SMPD3 [51]. Withanolide D triggers the apoptosis of leukemia MOLT-4 cells by upregulating SMPD3 expression after 15 min and downregulating after 45 min [52]. The oral administration of resveratrol suppresses intestinal tumorigenesis in mice and upregulates TSG101 mRNA expression (Table 1) [53]. All this warrants a detailed assessment exploring the roles of more natural-product-regulated cell functions in exosomal secretion in the future.

#### 2.2.2. Exosomal Assembly Effects of Natural Products in Regulating Cell Functions

Natural products also regulate exosomal assembly. *Astragalus membranaceus* extract enhances angiogenesis in myocardial infarction rats by upregulating vascular endothelial growth factor (VEGF), CD34, and endothelial nitric oxide synthase (eNOS) expression [54]. Moreover, D Rhamnose bhederin, a *Clematis ganpiniana*-derived bioactive component, inhibits exosome secretion from docetaxel-resistant breast cancer cells and alleviates the transmission of resistance [55]. D Rhamnose bhederin downregulates several miRNAs (miR-16-5p, miR-23a-3p, miR-24-3p, miR-26a-5p, and miR-27a-3p), in which miR-24-3p is predicted to target exosomal-assembly related genes, such as *STAM*, according to the miRDB database [31]. This warrants a detailed assessment exploring the roles of more natural-product-regulated cell functions in exosomal assembly in the future.

## 3. Prediction of the Targeting of Exosome-Processing and AKT-Signaling Genes of Certain Exosome miRNAs

Several exosomal miRNAs have been reviewed previously [4,16,57]. However, their potential impacts on exosomal processing (secretion and assembly) have rarely been investigated. In this study, utilizing the miRDB database [31], these exosomal miRNAs targeting exosome-processing genes (Section 2) were retrieved (Table 2). miR-29a-3p is predicted to target *SMPD3*. miR-101-3p and miR-21-5p/miR-30a-5p are predicted to target *RAB27A* and *RAB11A*, respectively. miR-6887-5p is predicted to target *RAB7A* and *RAB7B*. miR-142-3p and miR-24-3p are predicted to target *HGS* and *ATP13A2/STAM*, respectively. miR-106a-5p, miR-106b-5p, miR-21-5p, miR-223-3p, miR-365a-3p, and miR-374a-5p are predicted to target *MYO5B*. miR-522-3p and miR-8485 are predicted to target *PDCD6IP* (Table 2).

Additionally, miR-8485 is also predicted to target *SDC1* and *SDCBP*. miR-128-3p, miR-142-3p, miR-200c-3p, miR-223-3p, and miR-8485 are predicted to target *STAM*. miR-106a-5p and miR-106b-5p are predicted to target *TSG101*. Finally, miR-128-3p, miR-32-5p, and miR-92a-3p are predicted to target *VPS4B* (Table 2). Consequently, these examples demonstrate that many of the reported exosomal miRNAs have the potential to target exosomal processing genes. This warrants an advanced examination exploring the roles of exosomal processing genes in several reported exosomal miRNA studies in the future.

Moreover, some exosomal miRNAs are reported to regulate AKT signaling [58,59,60,61]. Among the examples of exosomal miRNAs listed in Table 2, miR-29a-3p is predicted to target AKT2 and AKT3 (Table 2). miR-374a-5p is predicted to target AKT1. miR-101-3p, miR-106a-5p, miR-106b-5p, miR-365a-3p, miR-6887-5p, and miR-30a-3p are predicted to target AKT1, ATK2, or AKT3. Those AKT1-, AKT2-, and AKT3-targeting exosomal miRNAs (miR-29a-3p, miR-374a-5p, miR-101-3p, miR-106a-5p, miR-106b-5p, miR-365a-3p, miR-6887-5p, and miR-30a-3p) also target some exosomal processing genes (Table 2).

Exosomal proteins can activate AKT signaling in the regulation of metastasis. Annexin A5, one of the exosome proteins in prostate cancer tissues, activates AKT signaling to stimulate the epithelial–mesenchymal transition (EMT) and upregulate matrix metalloproteinase-2 (MMP2) and MMP9 expression [5]. The exosomal miRNAs involved in AKT signaling have been applied in animal [59] and preclinical experiments [60]. Bone-marrow—mesenchymal-stem-cell-derived exosomes, which are rich in miR-126-3p (miR-126), enhance the migration and angiogenesis of human umbilical vein endothelial cells (HUVECs) [59]. This exosomal miR-126-3p stimulates vascularization at wound sites and improves cutaneous wound healing in mice models. Plasma exosomes isolated from Graves ophthalmopathy with an effective response to intravenous glucocorticoid therapy contain a high level of miR-885-3p, showing AKT inhibition and improving glucocorticoid sensitivity [60]. Consequently, the exosomal miRNAs with AKT modulating ability are potential tools for preclinical applications.

**Table 2:** Connecting some exosomal miRNAs to the predicted targets of exosome processing and AKT genes.

	ATP9A	ATP13A2	HGS	MYO5B	RAB27A	RAB11A	RAB7A	RAB7B	PDCD6IP	SDC1	SDCBP	SMPD3	STAM	TSG101	VPS4B	AKT
**miR-29a-3p** [4,16]												SMPD3				AKT2/3
**miR-101-3p** [4]					RAB27A											AKT3
**miR-106a-5p** [16,57]				MYO5B										TSG101		AKT3
**miR-106b-5p** [57]				MYO5B										TSG101		AKT3
**miR-128-3p** [57]													STAM		VPS4B	
**miR-142-3p** [4,16]			HGS										STAM			
**miR-200c-3p** [4,16]													STAM			
**miR-21-5p** [4,57]				MYO5B		RAB11A										
**miR-223-3p** [16]				MYO5B									STAM			
**miR-24-3p** [4,16]		ATP13A2											STAM			
**miR-32-5p** [57]															VPS4B	
**miR-365a-3p** [57]				MYO5B												AKT3
**miR-374a-5p** [57]				MYO5B												AKT1
**miR-522-3p** [57]									PDCD6IP							
**miR-6887-5p** [4]							RAB7A	RAB7B								AKT3
**miR-8485** [4]	ATP9A								PDCD6IP	SDC1	SDCBP		STAM			
**miR-92a-3p** [57]															VPS4B	
**miR-30a-3p** [62]						RAB11A										AKT3

The predicted targets for exosomal processing and AKT genes of the exosomal miRNAs were retrieved from the miRDB database (retrieval date: 12 November 2022).

## 4. Natural Products Modulate Exosome Production and Their Exosome Delivery for the Purpose of Cancer Treatment

Many natural-product-derived exosomes have been demonstrated to exhibit theranostic effects in cancer therapy [63,64,65,66]. The modulating results of exosome biogenesis and delivery by natural products are discussed as follows below.

Some natural products improve exosome biogenesis. Sulforaphane suppresses the fusion of early and late endosomes (GFP-Rab5a and GFP-Rab7a) with the lysosome, blocks the autophagy flux, promotes exosome production, and triggers exosome-dependent paracrine senescence by downregulating mTOR and transcription factor binding to IGHM enhancer 3 (TFE3) [67]. Sulforaphane induces a high protein concentration of exosomes and causes the accumulation of exosome marker CD63 in esophageal cancer cells. Moreover, supernatants from sulforaphane-treated cancer cells show high CD63 expression [67]. Consequently, sulforaphane triggers exosome biogenesis and secretion in esophageal cancer cells.

In contrast, some natural products suppress exosome biogenesis. Autophagy and lysosome dysfunction enhance exosome secretion [68,69] and vice versa. Asteltoxin inhibits mitochondrial ATP synthase and exosome generation by upregulating AMPK-dependent mTORC1 inactivation and lysosome activation [70]. Transmission electron microscopy analysis shows that asteltoxin induces lysosome–MVB fusion, causing the downregulation of exosome generation. Berberine suppresses the proliferation of colon cancer cells by downregulating acetyl-CoA carboxylase (ACC) for fatty acid synthesis and reducing exosome biogenesis and the secretion of colon and cervical cancer cells [71], an observation which is supported by the finding that berberine downregulates syntenin and TSG101, as intracellular vesicle markers.

Some natural-product-derived exosomes exhibit modulating effects on cell functions. Exosomes used in phytoagent deoxyelephantopin treatment, a plant deoxyelephantopin derivative, suppress the ROS-mediated proliferation of breast cancer cells, reversed by *N*-acetylcysteine [72]. Phytoagent deoxyelephantopin also enhances calcium-dependent exosome secretion from breast cancer cells. *Momordica charantia*-derived exosome-like nanovesicles suppress the proliferation and migration of glioma cells by downregulating phosphorylated PI3K/AKT [73]. *Fusobacterium nucleatum* is rich in colon cancer lesions associated with colon cancer carcinogenesis and metastases. Exosomes from *Fusobacterium nucleatum* enhance the invasion of colon cancer cells. This invasion is prevented by the bioactive compounds of *Paris polyphylla*, such as pennogenin 3-O-beta-chacotrioside and polyphyllin VI, which exhibit cell-killing effects on *Fusobacterium nucleatum* [74]. All this warrants an advanced examination exploring the impacts of exosomal biogenesis by natural products and natural-product-derived exosomes on cell functions in the future.

Moreover, exosomes are naturally generated, showing lower cytotoxicity and immunogenicity and higher biocompatibility than lipid-based nanoparticles [6,65,75]. Exosomes were reported to effectively deliver several natural products that can be exploited for preclinical anticancer therapy in vitro and in vivo [65]. The oral delivery of paclitaxel using milk-derived exosomes results in less side effects of immunologic toxicity and higher antitumor effects than i.v. in lung-tumor-xenograft nude mice [76]. Exosome-delivered curcumin exhibits a high in vitro stability and in vivo bioavailability [77]. Celastrol-loaded milk exosomes show a high degree of anti-lung tumor growth with in vivo biosafety [78]. This warrants the advanced testing of more natural products based on exosome delivery strategies in the future.

## 5. The Role of Natural-Product-Modulating miRNAs and Exosomes in Regulating Cancer Cell Functions 

A mounting array of literature reports that natural products modulate many miRNAs that regulate their target genes to affect several of the cell functions of cancer cells [79]. However, most of these studies did not investigate the impacts of exosomal miRNAs on anticancer effects using natural products.

Recently, several natural-product-induced exosomal miRNA studies have been reported. *Aurea helianthus* extract inhibits the migration and induces the senescence and autophagy of endometrial cancer cells [80]. Several miRNAs derived from the induced exosomes in these extract-treated endometrial cancer cells were upregulated or downregulated. However, there is a lack of systemic information on the modulating effects of drug-induced exosomal miRNAs based on natural products. Most natural-product-modulating miRNA studies have focused on impacts on cancer cell functions without considering the contribution of exosomes. Consequently, there are gaps between exosomal miRNAs and natural products in terms of their anticancer effects.

A total of 26 natural-product-modulated exosomal miRNAs that regulate cancer cell functions, such as antiproliferation (Section 5.1), apoptosis (Section 5.2), antimigration/anti-invasion/anti-EMT/anti-angiogenesis (Section 5.3), the modulation of chemo- and radio-resistance (Section 5.4), and others (Section 5.5), are summarized in Table 3. Many non-exosomal miRNA studies have assessed the impacts of the anticancer effects of natural products. In the future, a detailed examination of the roles of exosomes and investigations of their anticancer effects related to miRNAs and natural products should be carried out.

### 5.1. Antiproliferation by Natural-Product-Modulated Exosomal miRNAs 

Natural products may regulate cell proliferation by modulating miR-424-5p, miR-21-5p (miR-21), miR-101-3p, miR-1246, miR-155-5p (miR-155), miR-30a-5p, miR-34a-5p, and miR-30d-5p (Table 3), as described in the following section.

miR-424-5p is downregulated in breast cancer tissues. Resveratrol inhibits the proliferation of breast cancer cells by upregulating miR-424-5p and downregulating heterogeneous nuclear ribonucleoprotein A1 (HNRNPA1) [85] (Table 3).

Berberine, a natural alkaloid, inhibits the proliferation of multiple myeloma cells by downregulating miR-21-5p and upregulating its target, programmed cell death 4 (*PDCD4*) [86] (Table 3). The natural product butylcycloheptyl prodiginine promotes the antiproliferation of colon cancer cells by binding pre-miR-21-5p to inhibit the function of miR-21-5p [89]. Honokiol, a *Magnolia officinalis*-derived natural product, promotes the antiproliferation of osteosarcoma cells by downregulating miR-21-5p/AKT signaling [90]. Sophocarpine, a *Sophora flavescens*-derived bioactive compound, suppresses the proliferation of head and neck cancer cells by targeting miR-21-5p [92]. Dihydromyricetin, a natural flavonoid, suppresses the proliferation of cholangiocarcinoma cells by downregulating miR-21-5p [94]. Curcumin decreases the proliferation of liver cancer cells by downregulating miR-21-5p and upregulating its target, SRY-box transcription factor 6 (*SOX6*) [95] (Table 3).

Exosomal miR-101-3p displays tumor-suppressive and oncogenic functions. Oral cancer cells express low levels of miR-101-3p by targeting the collagen type X alpha 1 chain (*COL10A1*). In contrast, exosomes derived from bone marrow mesenchymal stem cells overexpress miR-101-3p to suppress oral cancer proliferation and migration [131]. miR-101-3p mimics suppress the proliferation and migration and trigger the apoptosis of medulloblastoma cells by targeting the enhancer of zeste homolog 2 (*EZH2*), a histone methyltransferase [132]. In contrast, exosomal miR-101-3p exhibits an oncogenic function to improve the proliferation and migration of colon cancer cells by downregulating its target, the homeodomain-interacting protein kinase (*HIPK3*) [133]. Berberine, a plant-bark-derived alkaloid, suppresses the proliferation of endometrial cancer cells by upregulating miR-101-3p to downregulate cyclo-oxygenase-2 (COX-2) [88] (Table 3).

Exosomal miR-1246 is reported to regulate cell migration. Exosomal miR-1246 from highly metastatic oral cancer cells promotes the migration and invasion of poorly metastatic oral cancer cells by downregulating the DENN/MADD-domain-containing 2D (DENND2D) [134]. In addition to antimigration, miR-1246 was reported to modulate proliferation (Table 3). Bladder cancer T24 cells highly express miR-1246. Curcumin inhibits the proliferation of bladder cancer cells by downregulating miR-1246 [98]. Combined treatment (curcumin and X-ray) synergistically suppresses its proliferation to a greater extent than individual treatments by decreasing miR-1246 expression [98].

Moreover, natural products were reported to modulate miR-155-5p, miR-30a-5p, and miR-34a-5p expression, regulating cancer cell proliferation (Table 3). Genistein, a soy isoflavone phytoestrogen, suppresses the proliferation of breast cancer cells by downregulating miR-155-5p [105]. Thymoquinone, a black-seed-oil-derived compound, suppresses liver fibrosis by upregulating miR-30a-5p to inhibit its target, such as snail family transcriptional repressor 1 (*SNAI1*), inhibiting EMT [111]. Norcantharidin, a cantharidin derivative, inhibits the proliferation of giant-cell tumors of the bone by upregulating miR-30a-5p and downregulating AKT, reversed by inhibiting miR-30a-5p [62]. Emodin, a natural anthraquinone derivative, suppresses liver cancer cell proliferation by upregulating miR-34a-5p [122].

Exosomal miR-30d-5p appears in higher levels in cervical cancer tissues than in normal controls [135]. However, the miR-30d-5p-modulating cell function has rarely been reported, particularly in regard to the antiproliferation of cancer cells. A recent study of natural products reported the antiproliferation effect achieved by the modulation of miR-30d-5p (Table 3). Piperlongumine, a long-pepper-derived amide alkaloid, suppresses the proliferation of osteosarcoma cells by downregulating miR-30d-5p and upregulating its target, the suppressor of cytokine signaling 3 (*SOCS3*) [130].

Furthermore, natural products may regulate cancer cell proliferation by modulating miR-200c-3p (Table 3). (−)-Sativan, a *Spatholobus suberectus*-derived isoflavane, suppresses the proliferation of breast cancer cells by upregulating miR-200c-3p to downregulate its direct target, such as prickle planar cell polarity protein 2 (*PRICKLE2*; *EPM5*) [109].

### 5.2. Apoptosis by Natural-Product-Modulated Exosomal miRNAs

Several natural products modulate apoptosis in cancer cells by downregulating miR-21-5p, miR-196a-5p (miR-196a), miR-210-3p, miR-365a-3p (miR-365), miR-34a-5p (miR-34a), miR-144-3p (miR-144), miR-23b-3p, and miR-382-5p (Table 3), as described in the following section.

Several natural products promote apoptosis through miR-21-5p in cancer cells. Ursolic acid triggers the apoptosis of glioblastoma cells by downregulating miR-21-5p [81] (Table 3). Resveratrol triggers the apoptosis of bladder cancer cells by downregulating miR-21-5p and AKT phosphorylation, reversed by miR-21-5p overexpression [83]. The downregulation of miR-196a-5p enhances cisplatin resistance [102]. Psoralen, a natural photosensitizing drug, triggers the apoptosis of gastric cancer cells to alleviate cisplatin resistance by upregulating miR-196a-5p and downregulating homeobox B7 (HOXB7) and HER2 expression [102]. Honokiol enhances the apoptosis of osteosarcoma cells by downregulating miR-21-5p/AKT signaling [90]. Dihydromyricetin triggers the apoptosis of cholangiocarcinoma cells by downregulating miR-21-5p [94]. PRP1, a *Platycodonis*-radix-derived polysaccharide, promotes the apoptosis of liver cancer cells by reducing miR-21-5p expression and inactivating AKT [100].

Exosomal miR-210-3p promotes the angiogenesis and tubulogenesis of endothelial cells [136] and enhances the metastasis of lung cancer cells [137]. A recent study demonstrated the novel function of the apoptosis-modulating effect of miR-210-3p, regulated by natural products. In hypoxic conditions, colon cancer cells enhance tumor progression. The transmission of hypoxic colon-cancer-cell-derived exosomal miR-210-3p to normoxic tumor cells prevents apoptosis and induces a protumoral effect [138]. 1΄S-1΄-Acetoxychavicol acetate (ACA), a wild ginger *Alpinia conchigera*-derived natural product, triggers the apoptosis of cervical cancer cells by downregulating miR-210-3p to upregulate its target, SMAD family member 4 (*SMAD4*) [113] (Table 3).

Exosomal miR-365a-3p regulates the chemoresistance of cancer cells involved in apoptosis. Exosomal miR-365a-3p derived from imatinib-resistant chronic myeloid leukemia (CML) cells provides drug resistance to, and prevents apoptosis in, sensitive CML cells [139]. 

Natural product studies showed that miR-365a-3p exhibits an apoptosis-modulating effect (Table 3). Crocin, a carotenoid pigment of saffron, induces cervical cancer cell apoptosis by upregulating Bax and downregulating BCL2 and miR-365a-3p [114]. The combination treatment of cervical cancer cells with crocin and cisplatin promotes antiproliferation and apoptosis by downregulating miR-365a-3p, an upregulator of BAX and BCL2 [140].

Exosomal miR-421 regulates the chemoresistance of cancer cells. Exosomes from cisplatin-resistant oral cancer patients enhance the proliferation and reduce the cisplatin sensitivity of cisplatin-resistant cells by downregulating miR-421 expression [141]. The hypermethylation of transcription-factor-activating-enhancer-binding protein 2e (TFAP2E) enhances 5-fluorouracil chemoresistance in gastric cancer cells by upregulating exosomal miR-421 [142]. However, the impact of apoptosis by exosomal miR-421 is unclear. A recent natural product study reported the apoptosis function through the modulation of miR-421 (Table 3). Isoliquiritigenin induces the apoptosis and DNA damage of oral cancer cells by downregulating miR-421 expression [116].

Exosomal miR-34a-5p regulates the proliferation of cancer cells. Normal fibroblasts exhibit higher miR-34a-5p levels than cancer-associated fibroblasts from oral cancer patients. miR-34a-5p overexpression in cancer-associated fibroblasts suppresses cancer cell proliferation and migration [143]. Exosomal miR-34a-5p induces the antiproliferation and apoptosis of pancreatic cancer cells [144]. Several natural product studies have demonstrated the apoptosis function of cancer cells through the modulation of miR-34a-5p (Table 3). Dihydroartemisinin triggers the apoptosis of prostate cancer cells by upregulating miR-34a-5p [120]. Isovitexin, a flavonoid, triggers the apoptosis of osteosarcoma cells by upregulating miR-34a-5p and downregulating BCL2 [121]. Crocin, a saffron-derived pigment, triggers the ROS-dependent apoptosis of papillary thyroid cancer cells by downregulating the miR-34a-5p and upregulating its target, protein tyrosine phosphatase non-receptor type 4 (*PTPN4*) [115]. 

miR-144-3p exhibits differential expressions in different cancer cells. miR-144-3p is downregulated in lung cancer cells. Exosomal miR-144-3p from bone marrow-derived mesenchymal stem cells suppresses the proliferation of lung cancer cells by downregulating cyclin E1 (CCNE1) and CCNE2 [145]. In contrast, miR-144-3p is upregulated in nasopharyngeal cancer cells. Exosomal miR-144-3p from nasopharyngeal cancer cells promotes angiogenesis [146]. However, the apoptosis-inducing effect of exosomal miR-421 has rarely been examined. Recent natural product studies demonstrated apoptosis induction through the modulation of miR-144-3p (Table 3). Berberine induces the apoptosis of lung cancer cells by upregulating miR-144-3p [87]. Licochalcone A, a *Glycyrrhiza inflata*-derived natural product, induces ER stress and the apoptosis of lung cancer cells by upregulating miR-144-3p [126].

miR-23b-3p suppresses the proliferation and migration of prostate [147] cancer cells, while it enhances pancreatic cell migration [148] and salivary cancer cell angiogenesis and metastasis [149]. However, the apoptosis function of exosomal miR-421 has rarely been reported. A natural product investigation validated the fact that apoptosis induction results from the modulation of miR-23b-3p (Table 3). 10-Hydroxycamptothecin, a *Nothapodytes nimmoniana*-derived natural product, causes the apoptosis of fibroblasts by upregulating miR-23b-3p [127].

Exosomal miR-382-5p from cancer-associated fibroblasts enhances the migration of oral cancer cells [150]. A recent natural product study demonstrated the novel function of apoptosis induction through the modulation of miR-382-5p (Table 3). Polydatin, a metabolite of trans-resveratrol, inhibits the proliferation and causes the apoptosis of colon cancer cells by upregulating miR-382-5p and downregulating its target, programmed cell death ligand 1 (*PD-L1*) [129]. 

### 5.3. Antimigration/Anti-Invasion/Anti-EMT/Anti-Angiogenesis by Natural-Product-Modulated Exosomal miRNAs

Several natural products regulate the migration, invasion, and angiogenesis of cancer cells by modulating miR-101-3p, miR-30a-5p, miR-34a-5p, miR-200c-3p, miR-21-5p, and miR-421 (Table 3), as described in the following section.

Some miRNAs showing migration-suppressing effects are upregulated by several natural products. Berberine suppresses the migration of endometrial cancer cells by upregulating miR-101-3p to downregulate cyclo-oxygenase-2 (COX-2) [88] (Table 3). Norcantharidin inhibits the migration of giant-cell tumors of the bone by upregulating miR-30a-5p and downregulating AKT, reversed by inhibiting miR-30a-5p [62]. Dihydroartemisinin suppresses the migration of prostate cancer cells by upregulating miR-34a-5p [120]. Honokiol, a *Magnolia grandiflora*-derived polyphenol, suppresses the leptin-promoted EMT of breast cancer cells by upregulating miR-34a-5p [91]. Anisomycin suppresses angiogenesis in ovarian cancer stem cells by upregulating miR-421 [117].

Similarly, exosomal miR-200c-3p suppresses the migration and invasion of lipopolysaccharide (LPS)-treated colon cancer cells by targeting zinc finger E-box-binding homeobox-1 (*ZEB-1*) [151]. The natural compounds enoxolone, magnolol, and palmatine chloride suppress the invasion of breast cancer cells by upregulating miR-200c-3p [108]. (−)-Sativan inhibits the migration of breast cancer cells by upregulating miR-200c-3p [109]. Similarly, curcumin, acting on colon cancer cells, exhibits the downregulation of EMT-related gene expression by upregulating miR-200c-3p and downregulating its target, *PRICKLE2* [96]. Isoliquiritigenin, a Glycyrrhizae Rhizoma-derived bioactive component, inhibits migration, metastasis, and breast tumor growth by inhibiting EMT and upregulating miR-200c-3p, which is downregulated in breast cancer tissues [110]. Ursolic acid, a pentacyclic triterpenoid, induces the apoptosis and inhibits the invasive ability of colon cancer cells by upregulating miR-200c-3p [82].

In contrast, some miRNAs showing migration-promoting effects are suppressed by natural product treatments. The transfer of hypoxic oral cancer exosomes containing miR-21-5p to normal cells improves their pro-metastatic effects [152]. Sophocarpine suppresses the epithelial–mesenchymal transition (EMT) of head and neck cancer cells by targeting miR-21-5p [92]. Dihydromyricetin inhibits the migration of cholangiocarcinoma cells by downregulating miR-21-5p [94]. Curcumin decreases the migration of liver cancer cells by downregulating miR-21-5p [95]. Sinomenine, a *Sinomenium acutum*-derived alkaloid, shows antimigration effects on lung cancer cells by suppressing miR-21-5p and MMP2/9 [101]. Asparanin A, a vegetable- and *Asparagus officinalis-derived natural product,* suppresses the migration of endometrial cancer cells by downregulating miR-421 [118].

### 5.4. Modulation of Chemo- and Radio-Resistance by Natural-Product-Modulated Exosomal miRNAs

Several natural products regulate migration, invasion, and angiogenesis in cancer cells by modulating miR-21-5p, miR-155-5p, miR-34a-5p, and miR-31-5p, as described in the following section. 

Some miRNAs showing resistance-promoting effects are downregulated by several natural products. miR-21-5p has been identified in exosomes from hypoxic oral cancer cells. miR-21-5p-containing hypoxic oral cancer exosomes also exhibit cisplatin resistance in oral cancer cells, as evidenced by exosome transfer experiments [153]. Natural products may inhibit drug resistance in cancer cells by downregulating miR-21-5p. Tricin, an *Allium atroviolaceum*-derived compound, sensitizes the docetaxel response to prostate cancer cells by downregulating miR-21-5p [93] (Table 3). 

Similarly, exosomal miR-155-5p enhances the migration or metastasis of gastric [154], lung [155], renal [156], and colon [157] cancer cells. Recently, a resistance-modulating function of exosome miR-155-5p was reported. Exosome miR-155-5p from oral cancer cells improves cisplatin resistance to cisplatin-sensitive cells by upregulating EMT [158]. A natural product study showed the resistance-modulating function of miR-155-5p (Table 3). (−)-Epigallocatechin gallate (EGCG), a green- or red-tea-derived bioactive compound, improves 5-fluorouracil (5-FU) sensitivity in colon cancer cells by suppressing miR-155-5p expression [106].

In contrast, some miRNAs showing resistance-suppressing effects are upregulated by several natural products. Rhamnetin and cirsiliol, the quercetin and flavonoid derivatives, enhance radiosensitization and suppress lung cancer cell EMT by upregulating miR-34a-5p [119]. miR-34a-5p is downregulated in liver cancer tissues. EGCG improves the radiosensitization of liver cancer cells by upregulating miR-34a-5p [107]. Similarly, bladder cancer tissues exhibit a low level of miR-31-5p. Mitomycin C sensitivity is enhanced in bladder cancer cells by upregulating miR-31-5p to target integrin α5 (*ITGA5*) [125].

### 5.5. Potential Modulation Effects of Target Immunotherapy of Cancer by Natural-Product-Modulated Exosomal miRNAs

The tumor immune microenvironment (TIME) comprises several types of immune cells. Some miRNAs were identified in the tumor-associated macrophages (TAM), natural killer (NK) cells, and myeloid-derived suppressor cells (MDSC) of TIME [159]. A comparison illustrated that some of them overlapped with the exosomal miRNAs modulated by natural products (Table 3). Upon inspection, some of the exosomal miRNAs (miR-21-5p, miR-200c-3p, miR-155-5p, miR-30a-5p, miR-34a-5p, miR-130a-3p, miR-101-3p, miR-142-3p, and miR-24-3p) listed in Table 3 were reported in certain immune cells of TIME [159], such as TAM, NK, and MDSC. However, the review in question [159] rarely mentioned the impacts of natural products.

Here, we discuss the indirect connections of these exosomal miRNAs to natural products (Table 4). TAM upregulates several exosomal miRNAs (miR-21-5p, miR-155-5p, miR-30a-5p, miR-101-3p, and miR-142-3p) but downregulates miR-34a-5p [159]. NK upregulates miR-155-5p, miR-130a-3p, miR-101-3p, and miR-24-3p. MDSC upregulates miR-21-5p, miR-200c-3p, miR-155-5p, and miR-30a-5p. Since some of the miRNAs listed in Table 4 are downregulated or upregulated by several natural products, their potential impacts in modulating the expressions of the TAM, NK, and MDSC of TIME are worthy of attention. Notably, TIME miRNAs that are not included in Table 3 and Table 4 may be modulated by natural products and, thus, require detailed investigation.

All this warrants a detailed assessment of all the miRNA-modulating effects of these natural products that are employed in cancer studies in the future.

### 5.6. Other Cell Functions Influenced by Natural-Product-Modulated Exosomal miRNAs

Several miRNAs, such as miR-31-5p, miR-3188, miR-24-3p, miR-30a-5p, miR-130a-3p, miR-142-3p, miR-30a-5p, and miR-382-5p, exhibit diverse effects other than the modulation of proliferation, apoptosis, migration, and resistance. Although these miRNAs modulate many functions, only a few natural products were retrieved from Pubmed and Google scholar.

Exosomal miR-31-5p regulates the proliferation and drug resistance of cancer cells. Macrophage-derived exosomal miR-31-5p enhances oral cancer cell proliferation by downregulating large tumor suppressor 2 (LATS2) [160]. Exosomal miR-31-5p from hypoxic lung cancer cells promotes metastasis [161]. Moreover, exosomal miR-31-5p is also involved in the regulation of drug resistance. Exosomal miR-31-5p enhances sorafenib resistance in renal cancer cells by targeting mutL homolog 1 (*MLH1*) [162]. Forkhead box C1 (FOXC1) functions as a transcriptional factor to promote the transcription of miR-31-5p and downregulate LATS2, leading to oxaliplatin resistance in colon cancer cells [163]. A natural product study showed that resveratrol alleviates 2,4,6-trinitrobenzenesulfonic-acid-solution (TNBS)-induced colitis by suppressing miR-31-5p expression to increase the number of regulatory T-cells [84] (Table 3).

Cancer-associated fibroblasts enhance the progression of head and neck cancer cells by downregulating exosomal miR-3188 [164]. Without considering exosomes, other cancer studies also reported the tumor suppressive function of miR-3188. miR-3188 inhibits the proliferation of nasopharyngeal [165] and lung [166] cancer cells by targeting *mTOR*. A natural product study showed that pinolenic acid, a *Pinus*-species-derived natural product, upregulates miR-3188 to target the pyruvate dehydrogenase Kinase 4 (*PDK4*) and the mitochondrially encoded ATP synthase membrane subunit 6 (*MT-ATP6*) genes, showing anti-inflammatory effects in rheumatoid arthritis patients [103] (Table 3).

Exosomal miR-24-3p modulates proliferation and drug resistance in cancer cells. Salivary exosomal miR-24-3p promotes the proliferation of oral cancer cells by targeting period circadian regulator 1 (*PER1*) [167]. Exosomal miR-24-3p from cancer-associated fibroblasts enhances methotrexate resistance and inhibits the apoptosis of colon cancer cells by suppressing caudal type homeobox 2 (CDX2) or hephaestin (HEPH) expression [168]. A natural product study showed that doxorubicin causes the miR-24-3p overexpression of the left ventricle [104]. Pachymic acid, a *Poria cocos*-derived natural product, alleviates doxorubicin-induced heart failure in rats by downregulating miR-24-3p [104] (Table 3). 

Exosomal miR-30a-5p (miR-30a) regulates several cell functions, such as chemoresistance and migration. Cisplatin-sensitive oral cancer cells exhibit higher miR-30a and lower Beclin 1 (BECN1) expression levels than cisplatin-resistant cells [169]. Exosomes from miR-30a-mimic-transfected cisplatin-resistant cells downregulate BECN1 and BCL2 expression to sensitize the cells to cisplatin. Vascular endothelial cells express exosomes containing higher miR-30a-5p levels than lung cancer cells. Exosomal miR-30a-5p derived from vascular endothelial cells suppresses the proliferation and migration of lung cancer cells by targeting cyclin E2 (*CCNE2*) [170]. Colon cancer mesenchymal stem cells are abundant in exosomal miR-30a-5p. This stem cell exosomal miR-30a-5p improves the proliferation and migration of colon cancer cells by targeting the MIA SH3 domain ER export factor 3 (*MIA3*) [171]. A natural product study showed that Nicotine causes the G1 arrest of periodontal ligament cells by upregulating miR-30a-5p to target *CCNE2* [95] (Table 3). 

Exosomal miR-130a-3p (miR-130a) regulates the proliferation and migration of cancer cells. Breast cancer tissues and plasma exosomes exhibit low miR-130a-3p levels. The overexpression of miR-130a-3p in breast cancer stem-cell-like cells suppresses proliferation and migration by targeting *RAB5B*, member of the RAS oncogene family (RAB5B) [172]. The serum of differentiated thyroid cancer patients shows low levels of exosomal miR-130a-3p, which upregulates its target, insulin-like growth factor 1 (*IGF-1*) [173]. Some natural product studies showed that miR-130a-3p possessed inflammation-related functions (Table 3). Kaempferol, a dietary flavonoid, suppresses the cytokine production of chondrocytes by upregulating miR-130a-3p and downregulating its targets, such as the signal transducer and activator of transcription 3 (*STAT3*) [123]. Chicoric acid, an Echinacea-derived natural product, reduces the LPS-induced inflammation of lung cancer cells by downregulating miR-130a-3p and upregulating IGF-1 [124] (Table 3).

miR-142-3p exhibits differential patterns in regulating proliferation in different cancer cells. An increase in miR-142-3p in oral cancer cells suppresses tumor-promoting changes in the recipient endothelial cells [174]. Exosomal miR-142-3p from monocytes can be transferred to retinoblastoma cells, inhibiting their proliferation [175]. In contrast, exosomal miR-142-3p may exhibit a proliferation-promoting effect. Exosomal miR-142-3p from HBV-infected liver cancer cells induces the ferroptosis of M1 macrophages to improve the proliferation of liver cancer cells [176]. Some natural product studies showed that miR-144-3p can modulate several cell functions, such as the regulation of proteasome, ER stress, and autophagy. Curcumin suppresses 20S proteasome activity in breast cancer cells by upregulating miR-142-3p and downregulating its target, such as the proteasome 20S subunit beta 5 (*PSMB5*) [97]. Licochalcone A, a *Glycyrrhiza inflata*-derived natural product, induces ER stress in lung cancer cells by upregulating miR-144-3p [126]. Berberine induces the autophagy of lung cancer cells by upregulating miR-144-3p [87] (Table 3).

Some natural product studies showed that miRNAs can modulate liver fibrosis. Thymoquinone, a black-seed-oil-derived compound, suppresses liver fibrosis by upregulating miR-30a-5p to inhibit its target, such as snail family transcriptional repressor 1 (*SNAI1*), suppressing EMT [111]. Astaxanthin, a xanthophyll carotenoid, inactivates liver-fibrosis-associated hepatic stellate cells by downregulating miR-382-5p [128]. This warrants a detailed evaluation of all the miRNA-modulating effects of the aforementioned natural products employed in cancer studies.

*Furthermore*, several exosomal miRNAs have been identified in a number of cancer cells, but no natural product studies have been reported to date. Some exosomal miRNAs show proliferation-/invasion-promoting effects. Exosomal miR-626 enhances the proliferation and migration of oral cancer cells by targeting nuclear factor I/B (*NFIB*) [177]. miR-10b-5p shows a higher expression in metastatic breast cancer cells than in non-metastatic breast cancer or normal cells. Exosomal miR-10b-5p transmission enhances the invasion capacity of normal breast cancer [178]. The delivery of exosomal miR-10b-5p from gastric cancer cells also improves the proliferation of fibroblasts [179].

In contrast, some exosomal miRNAs show proliferation-/invasion-suppressing effects. A vitamin D analog, eldecalcitol (ED-71)-induced exosomal miR-6887-5p, suppresses oral cancer cell proliferation by targeting the 3′-UTR of heparin-binding protein 17/fibroblast growth-factor-binding protein-1 (*HBp17*/*FGFBP-1*) [180]. Exosomal miR-3180-3p suppresses lung cancer proliferation and metastasis by targeting forkhead box P4 (*FOXP4*) [181]. 

### 5.7. Overview of the Natural Products and Their Modulating Exosomal miRNAs That Regulate Exosomal Processing

**Figure 3:**
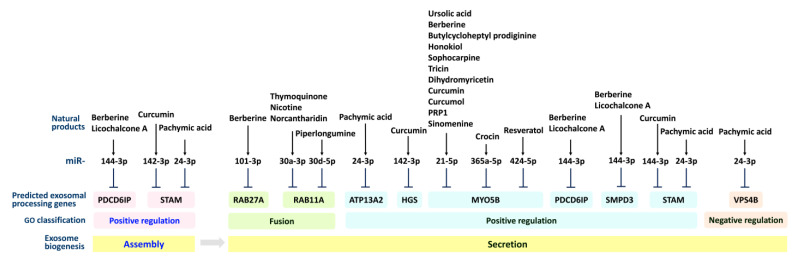
Overview of natural products modulating exosomal miRNAs through exosomal processing genes and exosomal assembly and secretion. The targets of exosomal processing genes with respect to natural-product-modulated miRNAs (Table 3) were predicted using the miRDB database. Some natural products (Table 3) are not shown here because the exosomal processing gene targets of their modulated miRNAs could not be identified in miRDB.

The connections of natural products with their exosomal miRNA-regulated cell functions are summarized in Table 3. However, the impacts of exosomal processing, their genes related to these natural products, and their modulated exosomal miRNAs remain unclear. Utilizing the miRDB database [31], the target prediction of the exosome-processing genes for these natural-product-modulated exosomal miRNAs (Table 3) was performed. From exosomal assembly to secretion, the exosomal processing genes targeted by natural-product-modulated exosomal miRNAs were plotted (Figure 3). This warrants a careful investigation of the predicted targets of these natural-product-modulated exosomal miRNAs based on experiments in the future.

## 6. Conclusions

Tumor-derived exosomes containing many biomolecules can regulate sophisticated cell functions. This review focused on our understanding of the roles of exosomal miRNAs in controlling cancer cell functions. The impacts of the modulating effects of natural products in regulating exosome processing and exosomal miRNAs were also summarized.

Many natural products exhibit diverse functions and affect the expression of many genes, but they the impacts of natural products on exosome biogenesis have been overlooked. By examining exosomal processing information derived from the GO database and PubMed/Google scholar searches, we noted that some of the altered genes belong to the classification of exosomal processing. Accordingly, this work represents a novel contribution to the study of the exosomal processing of natural products.

Similarly, many exosomal miRNAs have been reported but lack detailed investigations of their regulation of exosomal processing. By utilizing the miRDB database, the potential impacts of exosomal processing genes were predicted to be targeted by exosomal miRNAs. This prediction further provides a direction for future research, which should aim to assess the detailed mechanisms of exosomal miRNAs, although further experiments are still required to confirm them.

Finally, we collected and organized several natural products and their associated modulations of exosomal miRNAs and cell functions, such as proliferation, apoptosis, migration, the tumor immune microenvironment, and other diverse effects. The potential roles of exosomal processing in these natural product investigations were further assessed using information retrieved from the miRDB database. Similarly, we demonstrated that some natural-product-modulated exosomal miRNAs overlap with tumor-immune-microenvironment-associated miRNAs. Although they are indirectly connected, this information provides a future direction for research, which should aim to validate whether these natural products can modulate exosomal miRNAs to regulate the tumor immune microenvironment. 

Consequently, we offer a clear conclusion that several exosome-processing genes involved in exosomal secretion and assembly are organized in connection to natural products based on our utilization of the miRDB database to retrieve the target predictions of exosomal miRNAs. Accordingly, we filled the gaps in current knowledge between the exosomal processing of exosomal miRNAs and natural products. 

Notably, the miRDB-database-predicted targets of exosomal processing genes were collected based on different cell types. Different cell types may show various miRNAs and targeting responses. This warrants careful examination based on wet experiments to validate the relationship between exosomal miRNAs and natural products in order to explore their impacts on the modulation of cancer cell functions. 

This review sheds light on the connections between exosomes, exosomal miRNAs, natural products, and cancer cell functions, providing a clear direction for future research on the modulation of exosomal miRNAs by natural products.

## Figures and Tables

**Table 3:** Connecting natural products to exosomal miRNA-regulated cell functions.

Natural Products	miRNAs	Cell Functions	Cancer	References
Ursolic acid	↓miR-21-5p	apoptosis	glioblastoma	[81]
	↑miR-200c-3p	apoptosis, anti-invasion	colon	[82]
Resveratrol	↓miR-21-5p	apoptosis	bladder	[83]
	↓miR-31-5p	anticolitis	(T cells)	[84]
	↑miR-424-5p	antiproliferation	breast	[85]
Berberine	↓miR-21-5p	antiproliferation	myeloma	[86]
	↑miR-144-3p	apoptosis, autophagy	lung	[87]
	↑miR-101-3p	antiproliferation, antimigration	endometrial	[88]
Butylcycloheptyl prodiginine	↓miR-21-5p	antiproliferation	colon	[89]
Honokiol	↓miR-21-5p	apoptosis	osteosarcoma	[90]
	↑miR-34a-5p	anti-EMT	breast	[91]
Sophocarpine	↓miR-21-5p	antiproliferation, anti-EMT	head/neck	[92]
Tricin	↓miR-21-5p	chemosensitization	prostate	[93]
Dihydromyricetin	↓miR-21-5p	antiproliferation, antimigration	cholangiocarcinoma	[94]
Curcumin	↓miR-21-5p	antiproliferation, antimigration	liver	[95]
	↑miR-200c-3p	anti-EMT	colon	[96]
	↑miR-142-3p	20S proteasome suppression	breast	[97]
	↓miR-1246	antiproliferation	bladder	[98]
Curcumol	↓miR-21-5p	antiproliferation	colon	[99]
PRP1	↓miR-21-5p	apoptosis	liver	[100]
Sinomenine	↓miR-21-5p	antimigration	lung	[101]
Psoralen	↑miR-196a-5p	apoptosis	gastric	[102]
Pinolenic acid	↑miR-3188	anti-inflammation	(rheumatoid arthritis)	[103]
Pachymic acid	↓miR-24-3p	anti-heart failure	(left ventricle)	[104]
Genistein	↓miR-155-5p	antiproliferation	(cardiac)	[105]
(−)-Epigallocatechin gallate	↓miR-155-5p	chemosensitization	colon	[106]
	↑miR-34a-5p	radiosensitization	liver	[107]
Enoxolone, Magnolol,	↑miR-200c-3p	anti-invasion	breast	[108]
Palmatine chloride				
(−)-Sativan	↑miR-200c-3p	apoptosis, antimigration	breast	[109]
Isoliquiritigenin	↑miR-200c-3p	antimigration	breast	[110]
Thymoquinone	↑miR-30a-5p	anti-liver fibrosis	(liver)	[111]
Nicotine	↑miR-30a-5p	G1 arrest	(periodontal ligament)	[112]
Norcantharidin	↑miR-30a-5p	antiproliferation, antimigration	giant cell tumor of bone	[62]
1΄S-1΄-acetoxychavicol acetate	↓miR-210-3p	apoptosis	cervical	[113]
Crocin	↓miR-365a-3p	apoptosis	cervical	[114]
	↓miR-34a-5p	apoptosis	papillary thyroid	[115]
Isoliquiritigenin	↓miR-421	apoptosis, DNA damage	oral	[116]
Anisomycin	↑miR-421	anti-angiogenesis	ovarian	[117]
Asparanin A	↓miR-421	antimigration	endometrial	[118]
Rhamnetin, Cirsiliol	↑miR-34a-5p	radiosensitization, anti-EMT	lung	[119]
Dihydroartemisinin	↑miR-34a-5p	apoptosis, antimigration	prostate	[120]
Isovitexin	↑miR-34a-5p	apoptosis	osteosarcoma	[121]
Emodin	↑miR-34a-5p	antiproliferation	liver	[122]
Kaempferol	↑miR-130a-3p	cytokine reduction	(chondrocyte)	[123]
Chicoric acid	↓miR-130a-3p	anti-inflammation	lung	[124]
Mitomycin C	↑miR-31-5p	chemosensitization	bladder	[125]
Licochalcone A	↑miR-144-3p	ER stress, apoptosis	lung	[126]
10-Hydroxycamptothecin	↑miR-23b-3p	apoptosis	(fibroblast)	[127]
Astaxanthin	↓miR-382-5p	anti-liver fibrosis	(liver)	[128]
Polydatin	↑miR-382-5p	apoptosis	colon	[129]
Piperlongumine	↓miR-30d-5p	antiproliferation	osteosarcoma	[130]

↑, enhance or activate; ↓, inhibit or inactivate. Non-cancer studies are shown in parentheses.

**Table 4:** Connecting natural-product-modulated exosomal miRNAs to TIME.

miRNAs	miRNAs Status in TIME	miRNA Effects of Natural Products *
miR-21-5p	↑TAM [159] ↑MDSC [159]	↓ miR-21-5p (Ursolic acid [81], Resveratrol [83], Berberine [86], Butylcycloheptyl prodiginine [89], Honokiol [90], Sophocarpine [92], Tricin [93], Dihydromyricetin [94], Curcumin [95], Curcumol [99], PRP1 [100], Sinomenine [101])
miR-200c-3p	↑MDSC [159]	↑ miR-200c-3p (Urolic acid [82], Curcumin [96], Enoxolone, Magnolol, Palmatine chloride [108], (−)-Sativan [109], Isoliquiritigenin [110])
miR-155-5p	↑TAM,↑MDSC,↑NK [159]	↓ miR-155-5p (Genistein [105], (−)-Epigallocatechin gallate [106])
miR-30a-5p	↑TAM,↑MDSC [159]	↑ miR-30a-5p (Thymoquinone [111], Nicotine [112], Norcantharidin [62])
miR-34a-5p	↓TAM [159]	↑ miR-34a-5p (Honokiol [91], (−)-Epigallocatechin gallate [107], Rhamnetin, Cirsiliol [119], Dihydroartemisinin [120], Isovitexin [121], Emodin [122])
		↓ miR-34a-5p (Crocin [115])
miR-130a-3p	↑NK [159]	↑ miR-130a-3p (Kaempferol [123])
		↓ miR-130a-3p (Chicoric acid [124])
miR-101-3p	↑TAM [159]	↑ miR-101-3p (Berberine [88])
miR-142-3p	↑TAM [159]	↑ miR-142-3p (Curcumin [97])
miR-24-3p	↑NK [159]	↓ miR-24-3p (Pachymic acid [104])

* These natural products and their modulated exosomal miRNAs were collected from Table 3. The literature [159] did not provide information on natural products. ↑, enhance; ↓, inhibit. Tumor immune microenvironment (TIME); tumor-associated macrophages (TAM); natural killer (NK); myeloid-derived suppressor cells (MDSC).
